# Addition of Arbuscular Mycorrhizal Fungi Enhances Terpene Synthase Expression in *Salvia rosmarinus* Cultivars

**DOI:** 10.3390/life13020315

**Published:** 2023-01-23

**Authors:** Emily Leggatt, Alistair Griffiths, Simon Budge, Anthony D. Stead, Alan C. Gange, Paul F. Devlin

**Affiliations:** 1 Department of Biological Sciences, Royal Holloway University of London, Egham TW20 0EX, UK; 2 Science Department, Royal Horticultural Society, Woking GU23 6QB, UK; 3 Vitacress Herbs, Chichester PO20 1LJ, UK

**Keywords:** arbuscular mycorrhizal fungi, commercial herb cultivation, gene expression, rosemary, terpene synthase, volatile production

## Abstract

Culinary herbs are commercially cultivated for their wide range of volatile compounds that give characteristic aromas and tastes. Rosemary (*Salvia rosmarinus* Spenn.) is an excellent model for assessment of methods improvement of volatile production as cultivars offer a wide variety of aromatic profiles due to the large family of terpene synthase genes. Arbuscular mycorrhizal fungi (AMF) associations have been shown to improve essential oil production in aromatic plants and offer one approach to enhance aroma in commercial herb production. Changes in the expression of seven different terpene synthases were compared in six rosemary cultivars in response to addition of AMF to a peat substrate. Addition of AMF profoundly influenced terpene synthase expression in all cultivars and did so without impacting the optimised plant size and uniformity achieved in these conditions. In addition, two methods for AMF application, developed with the horticultural industry in mind, were tested in this study. Uniform incorporation of AMF mixed into the growing substrate prior to planting of a root plug produced the most consistent root colonisation. Overall, our findings demonstrate the potential for the use of AMF in the improvement of aroma in culinary herbs within a commercial setting but show that outcomes are likely to greatly vary depending on variety.

## 1. Introduction

Culinary herbs are commercially grown across the world for their wide range of volatile compounds that give characteristic aromas and tastes. Leading commercial growers are located globally and freshly grown herbs represent 5% of the global horticulture market with a predicted revenue of USD 1183.2 million between 2018 and 2025 [1]. There is a growing demand by consumers for high quality fresh herbs in cooking, leading to increasing potted herbs sales with consumers favouring plants with strong aromas and taste. As a result, improvements to the growth, aroma and taste are sought after by horticultural growers to meet with consumer demands.

Aromatic volatiles are phenolic terpenoid compounds synthesised in plants through two main cellular pathways: the plastidial methylerythritol 4-phosphate (MEP) pathway and the cytosolic mevalonate (MVA) pathway. All terpenoids are derived from the precursors dimethylallyl pyrophosphate (DMAPP) and isoprene diphosphate (IPP) formed during the later catalytic steps of both pathways [2]. IPP and DMAPP are further condensed by prenyltransferases, resulting in intermediates of different chain lengths and cyclization [3]. These intermediates are then used by terpene synthases to produce aromatic volatiles. The diversity of terpenoids produced is attributed to the enzymatic activity of these pathways but also to the large family of multi-substrate terpene synthases [4]. Altering the expression of terpene synthases in planta would likely alter the aromatic profile.

Rosemary (*Salvia rosmarinus* Spenn.) is a Mediterranean shrub belonging to the *Lamiaceae* Martinov family and is commonly cultivated for the active properties of its essential oils. It is an excellent model for assessment of methods looking to improve volatile production in potted culinary herbs. The antioxidant, antimicrobial, aromatic and preservative properties obtained from the leaves make rosemary valuable to both the pharmaceutical and food industries. Rosemary is widely cultivated for the fresh herb industry as a potted plant for culinary use. The main volatile constituents of rosemary that contribute to aroma are α-pinene, linalool, cymene, and eucalyptol [5] among others, and are all synthesised by terpene synthases. In the family *Lamiaceae*, there is a large chemo-diversity of terpene synthases, as studied in sweet basil *(Ocimum basilicum* L.), sweet marjoram (*Origanum majorana* L.), oregano (*Origanum vulgare* L.) and rosemary [6]. This diversity in terpene synthases provides the basis for breeding rosemary cultivars with desirable aromas by using gene markers for selected terpene synthases. The horticultural industry also seeks improvements to current rosemary cultivars on the market without the need for extensive breeding programmes. The following trials have been conducted to investigate the effects arbuscular mycorrhizal fungi (AMF) have on the volatile production in current horticultural rosemary cultivars.

The addition of AMF has been shown to improve the volatile production in rosemary [7]. Previous studies have generally investigated the effect of AMF bio-stimulants on rosemary growth and aroma while under environmental stressors, such as high salinity, drought, or nutrient deficiencies, as these are the challenges that face field grown rosemary crops [8,9]. In glasshouse herbs, such as basil, the addition of fungal bio-stimulants to the substrate mitigated the effects of salinity stress and improved the production of aromatic products in the plant [10]. However, another study conducted by Saia et al. [10] investigated the effect of AMF bio-stimulants in plants that were not saline stressed. They found the antioxidant content in basil increased with AMF addition through increases in rosmarinic acid production [10].

The addition of fertilizers (inorganic and organic) to the growth substrate of rosemary revealed a positive correlation between total terpene compounds and the nitrogen and phosphorus content in leaves [11]. It was suggested that the increased availability of nitrogen for terpene synthase activity, and the availability of phosphorus for precursors in the MEP pathway increased aromatic volatile production. Bustamante et al. [11] also reported that the fertilisation effects are a direct nitrogen trade-off between growth and the terpenoid pathways by which volatiles are synthesised. Therefore, increasing fertilization throughout the growth of the crop may not directly benefit the terpenoid pathway. Mycorrhizal associations in rosemary have previously been found to enhance growth and antioxidant properties of the leaves through increases in carnosol, ferulic acid, asiatic acid and vanillin [12]. It is known that mycorrhizal associations improve growth in plants and can improve production of specialised metabolites [13]. It is less well understood how AMF influence gene expression in specialised metabolism, and, in particular, terpene synthase expression.

One of the main challenges facing the horticultural industry is to find solutions that improve the growing conditions and the aromatic profiles of the plants. In addition, there is a need in horticulture for environmentally conscious and sustainable solutions whereby there can be a reduced use of chemical sprays and fertilizers. Mycorrhizal fungal associations with the plant can provide part of a solution to sustainable horticulture as they enhance metabolite production by increasing plant nutrient uptake. The aim of this investigation was to assess the effect of beneficial mycorrhizae on gene expression of terpene synthases for improved volatile production in horticultural rosemary. The effect of AMF on physiology and terpene synthase expression among different rosemary cultivars was investigated to better understand how cultivars respond to AMF in terms of growth and aroma production.

## 2. Materials and Methods

### 2.1. Propagation of Rosemary Cuttings

Semi-hard wood cuttings 8 cm long were taken from six cultivars of rosemary, namely, ‘Bolham Blue’; ‘Blue Boy’; ‘Logee Blue’; and ‘Roman Beauty’, sourced from Downderry Nursery, Tonbridge, UK; ‘Perigord’ obtained from Vitacress Herbs, Runcton, UK; and ‘Vatican Blue’ obtained from Jekka’s, Bristol, UK. The top leaves were left on the cutting and a sharp scalpel was used to remove excess leaves and cut below an internode. The cuttings were rooted in TPS peat substrate mix (Jiffy Products International, Moerdijk, The Netherlands). They were maintained in propagating boxes at 18 °C with 12 h warm white fluorescent light (120 mmol m^−2^ s^−1^, 12 h dark cycles of for 2–3 weeks until roots were produced. The cuttings were kept moist during root growth using a spray bottle. The cuttings were re-potted once rooted in 0.4 L pots filled with TPS peat substrate mix.

### 2.2. AMF Trials Design

The trial was conducted at the Vitacress commercial glasshouse in Runcton, UK, under controlled ambient conditions, an average temperature of 20.2 °C (±0.8 in the day and average temperature of 18.3 °C (±0.9) during the night over nine weeks. The experiment was carried out from February to April 2020, with natural light supplemented by SON-T high pressure sodium lamps (6000 lux) when the natural light was less than 8000 lux to maintain a light schedule of 12 h light/dark cycle. Pots were irrigated with potable water as required to keep the substrate moist. The AMF mixture used was the RGPRO HORTI 2 (PlantWorks Ltd., Sittingbourne, UK) mixture composed of five AMF species: *Funneliformis mosseae*, *Funneliformis geosporus, Claroideoglomus claroideum, Rhizophagus intraradices,* and *Glomus microaggregatum*. Two AMF application methods were used. The direct application method (Method 1) involved 5 g of AMF mixture added directly to the planting hole of each pot at the repotting stage, then the rosemary root plug was planted directly into the hole containing AMF. The AMF mixture application method (Method 2) involved 25 g of AMF mixture mixed into the peat-based compost, and dispersed evenly, per pot prior to planting the root plug. The control condition was peat substrate without the AMF mixture. In Trial 1, the cultivar Perigord was grown with both AMF application methods. Twelve pots were used as a control of just peat substrate, 12 pots were treated with the direct application (Method 1), and 26 pots were treated with an AMF mixture (Method 2). Plants were grown under controlled conditions for 64 days. In Trial 2, five rooted plugs of each cultivar were potted in peat substrate containing the AMF mixture and were grown for 64 days in controlled conditions. At the end of the trials, random sampling of six plants was performed to take measurements of plant height, plant width, fresh weight of the leaves and stems, dry weight of leaves and stems, total phenolic and total antioxidant content of the leaves. Root samples from five plants were taken from each substrate condition to assess AMF colonization.

### 2.3. Plant Growth Measurements

Plant growth was assessed to investigate any changes after AMF addition. The fresh and dry weight of the plants was recorded to assess any growth difference by measuring the total biomass of the stems and leaves of the plants. Height and width measurements were taken in triplicate from images of plants from the experimental conditions of each cultivar using automated measuring via image analysis software, ImageJ [14]. Six plant replicates of ‘Perigord’ were used to measure the fresh and dry weight of the stems and leaves. The fresh weight was measured after separating the stems from the roots and the stems were weighed. The dry weight was measured after air drying the stems at 50 °C in a drying cabinet for three days.

### 2.4. Total Antioxidant Content and Phenolic Content Assays

Total phenolic content was determined by the Folin-Ciocalteu (F-C) assay, as modified by Sánchez-Rangel et al. [15]. Plant extracts were prepared by grinding 0.2 g of rosemary leaf tissue in 2 mL of Acetate buffer (1.6% Acetic acid and 6% Sodium Acetate Trihydrate) with a pestle and mortar. The extract was transferred to a 1.5 mL microcentrifuge tube and centrifuged at 8000× *g* for five minutes. The supernatant was then used either directly in the assay or diluted with acetate buffer. The dilution factor was accounted for during data analysis. The assay solution consisted of 17.84 mM F-C reagent (Sigma-Aldrich, Gillingham, UK) and 71.39 mM sodium carbonate in distilled water. A total of 300 µL of reagent was added to 15 µL of the extracted sample into a well of a 96 well microtiter plate and incubated for 2 h. Gallic acid half serial dilutions from 1 mM stock were used as a standard. The absorbance was read using a plate-reading spectrophotometer (Spectramax Plus 384 Microplate Reader, Molecular Devices LLC, Wokingham, UK) at 765 nm. The Gallic Acid Equivalent (GAE) was calculated from the absorbance and expressed in mg of GAE/g of fresh weight tissue.

A ferric reducing antioxidant power assay was used to determine the antioxidant capacity of rosemary extracts in ascorbic acid equivalents. Each reagent was prepared fresh. Reagents included 25 mL of 50 mmol/L acetate buffer at pH 3.6, 10 mmol/L of 2,4,6-tripyridyl-s-triazine (Sigma-Aldrich, UK) dissolved in 2.56 mL 40 mmol/L HCl and 20 mmol/L of Ferric Chloride Hexahydrate dissolved in 25 mL distilled water. A total of 300 µL of this solution was then pipetted into a 96-well microtiter plate containing 30 µL of the extracted samples. The absorbance was read immediately using a plate-reading spectrophotometer (Spectramax Plus 384 Microplate Reader, Molecular Devices LLC, UK) at 595 nm. The Ascorbic Acid Equivalent (AAE) was then calculated in mg of AAE/g of fresh weight leaf tissue.

### 2.5. Root Staining and Microscopy

The roots from the rosemary plants treated with the two AMF application methods were stained for fungal structures and observed under a microscope to estimate percentage colonisation. The following protocol was adapted to clear the roots of rosemary and stain AMF structures within the roots of the AMF treated plants. The roots of un-supplemented plants were also stained for AMF presence as a control. A total of 5 g of root material was taken from the plant and washed in water to remove the substrate. Roots were placed in a 50 mL Falcon tube with 50 mL of 10% *w/v* KOH and given a heat pre-treatment of 60 °C in a water bath for 1 h. The roots were left to clear at room temperature for 24 h. The cleared roots were rinsed and 5% *v/v* HCl was added to the roots for 1 min. The HCl solution was removed and the stain trypan blue was added (0.01% trypan blue (Sigma-Aldrich, UK), 2.5% acetic acid, 50% glycerol). Whole roots were measured then mounted on microscope slides and observed under a compound microscope. Colonization was quantified using the cross-hair eyepiece method [16,17].

### 2.6. RNA Extraction from Rosemary Leaves

RNA extraction was performed using the Qiagen RNeasy Plant Mini Kit (Qiagen, Germantown, MD, USA). Initial leaf tissue disruption was carried out by grinding leaf samples in liquid nitrogen with a pestle and mortar. An alternative lysis buffer was used to improve RNA yield. The lysis buffer was developed by [18] for plant tissues with high levels of phenolics and polysaccharides. The lysis buffer consisted of 4 M guanidine isothiocyanate, 0.2 M sodium acetate at pH 5.0, 25 mM PVP-40 (polyvinylpyrollidone, Sigma-Aldrich, UK) and 1% final concentration of *β*-mercaptoethanol (Sigma-Aldrich, UK) was added immediately before use. A total of 20% sarkosyl (Sigma-Aldrich, UK) was added to the sample lysate before incubating at 70 °C for 10 min. The Qiagen protocol was followed as instructed in the kit. The extracted RNA was quantified using a NanoDrop spectrophotometer (Thermo Scientific, Waltham, MA, USA). RNA integrity was checked by gel electrophoresis, with RNA gel loading dye (Thermo Scientific, MA, USA) and an agarose gel with TAE as the buffer and stained with ethidium bromide. Bands were visualised under UV light with the NuGenius (Syngene, Cambridge, UK) imaging system.

### 2.7. Reverse Transcription and Quantitative PCR

Reverse transcription of extracted RNA was performed with the QuantiTect Reverse Transcription Kit (Qiagen, Maryland). gDNA was removed as per instructions, then 50 µL per sample was reverse transcribed at a final concentration of 1 µg of RNA. The cDNA synthesis was performed in a Techne 5-prime thermocycler (Cole-Parmer Ltd., Saint Neots, UK) with the following temperatures: 42 °C for 60 min, followed by 70 °C for 10 min and a final hold of 4 °C. qPCR was performed on a Rotorgene 6000 (Qiagen, MD, USA) using the primer sequences in Table 1 and using GAPDH as the housekeeping gene. Three biological replicates, chosen at random from the trials, and two technical replicates were used. 0.5 µg of cDNA per sample was pipetted along with 1X SYBR Green master mix (QuantiTect SYBR Green RT-PCR kit from Qiagen, MD, USA) with each primer set at a final concentration of 200 nM. A QIAgility robot (Qiagen, MD, USA) automated pipette was used for pipetting accuracy. The following temperature programme was used for qPCR: an initial denaturing of 94 °C for 2 min, followed by a cycle of 94 °C for 15 s, 58 °C for 45 s and 72 °C s for 30 cycles. Cycle threshold values were used to calculate relative expression using the ∆∆Ct method [19].

### 2.8. Statistical Analysis

For comparison of the two application methods of AMF, statistical analysis was conducted by one-way ANOVA in R Studio version 1.4.1106 [20]. The data were tested for normality of residuals and Levene’s test for equal variances were *p* ≤ 0.05. Two-way ANOVA with Tukey multiple comparisons of means post hoc was performed on the cultivar comparisons. The dependent factors were height of stems, width of stems, total antioxidant content, total phenolic content, dry weight of stems, fresh weight of stems and relative gene expression. Each dependent factor was analysed separately, the independent factors in each case were the presence/absence of AMF and the rosemary cultivars.

## 3. Results

### 3.1. AMF Addition to the Substrate Increases Phenolic Content and Alters Gene Expression of Terpene Synthases without Altering the Biomass or Morphology of Rosemary ‘Perigord’

**Figure 1:**
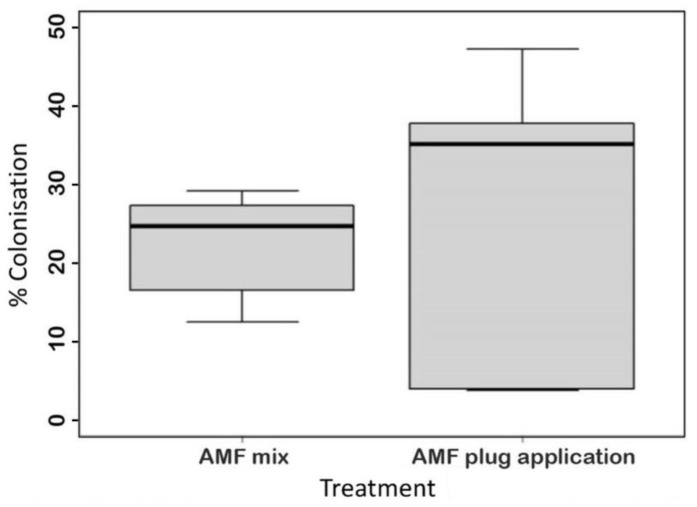
Observations of Arbuscular mycorrhizal fungi colonization in the roots of rosemary ‘Perigord’. Mean percentage colonization of two AMF treatments; an AMF mixture added to the substrate and a direct application to the root plug.

The addition of AMF to the substrate of the current commercial cultivar, ‘Perigord’, was trialled to assess the change in specialised metabolite production and aromatic content. Two different AMF application methods were trialled, one was a direct application to the root plug, the second method was a mixture of AMF into the substrate. Colonisation was observed with both application methods while no mycorrhizal colonisation was detected in any of the control plants. Microscopic observation of AMF root colonisation demonstrated no statistical difference between the levels of colonisation in each application method; however, the plug application method showed more variable colonisation (Figure 1).

**Figure 2:**
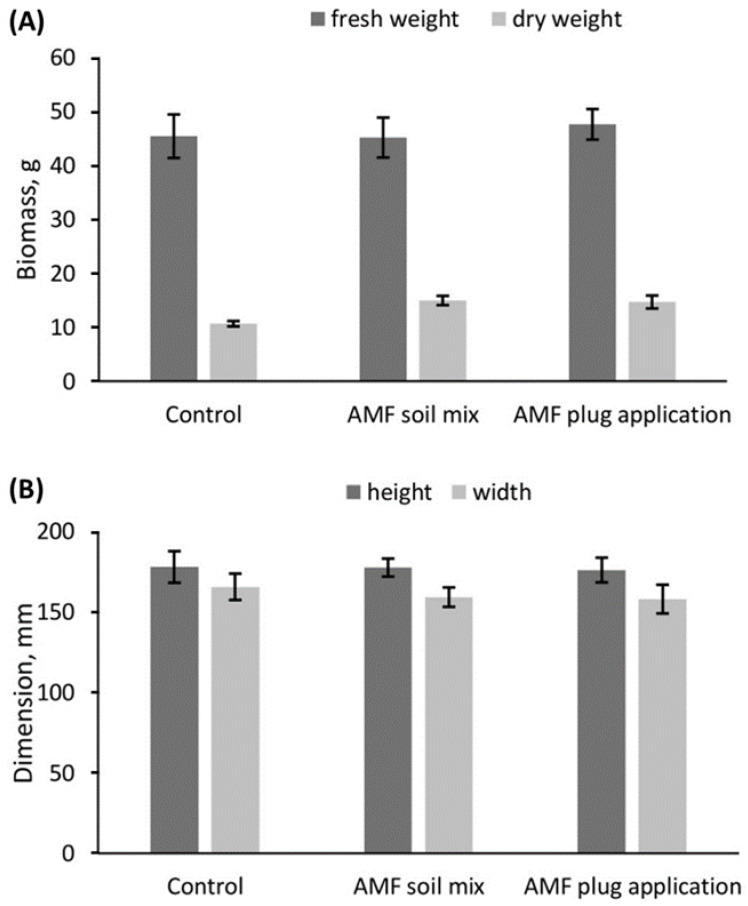
Growth assessment of rosemary ‘Perigord’ grown in peat substrate with additional Arbuscular Mycorrhizal Fungi applied via two different application methods. (**A**) Fresh weight and dry weight of the stems. (**B**) Height and width measurements of the plants. Error bars are SEM for each AMF condition.

The addition of AMF with either method had no significant effect on fresh and dry weight of rosemary plants (Figure 2A) or on the height and width of the plants (Figure 2B and Supplementary Figure S1).

**Figure 3:**
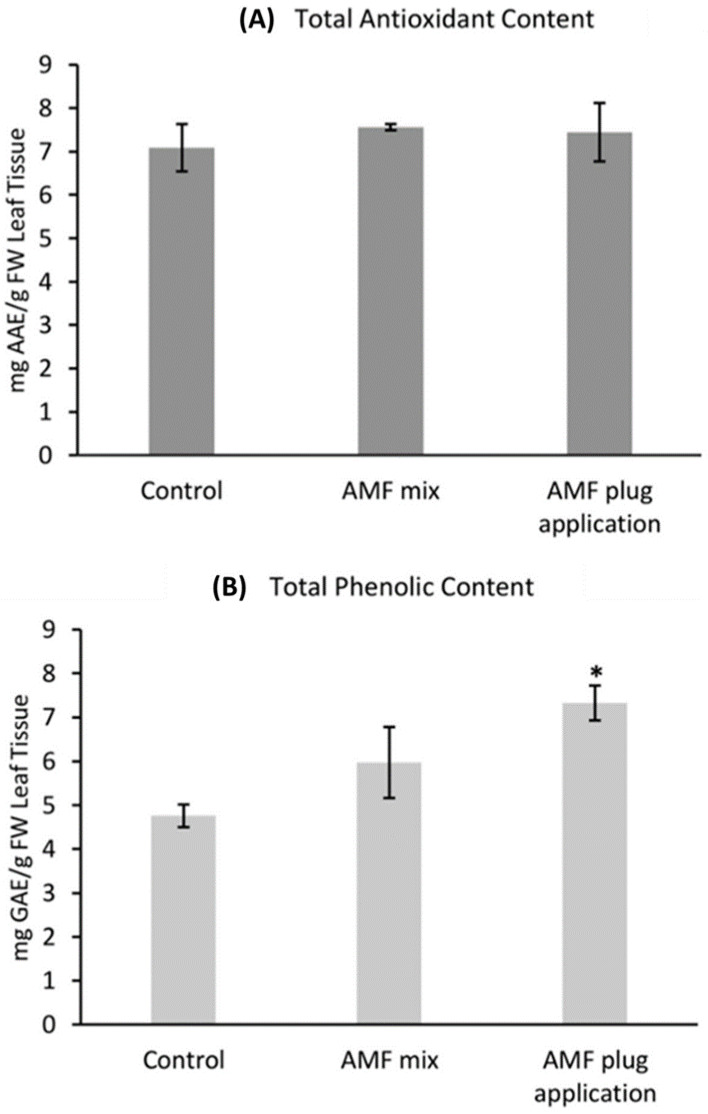
Specialised metabolite production in rosemary ‘Perigord’ treated with two Arbuscular mycorrhizal fungi AMF application methods. (**A**) Antioxidant content expressed as ascorbic acid equivalent (AAE). (**B**) Phenolic content expressed as gallic acid equivalent (GAE). Mean of treated plants with * above are significantly different to the control (*p* < 0.05 based on the Tukey HSD-test). Error bars are SEM of each treatment group.

Both application methods had no effect on the total antioxidant content of the plants (F_2,6_ = 0.21, *p* > 0.05, Figure 3A). However, a FRAP assay showed a clear increase in phenolic content by 33% for ‘Perigord’ treated with AMF mix and by 54% for ‘Perigord’ treated with the AMF root plug (Figure 3B). Statistical analysis, however, showed this was only significant in the case of the plug application method (F_2,6_ = 3.76, *p* < 0.05). The AMF mixture showed no significant difference in the phenolic content (Figure 3B).

**Figure 4:**
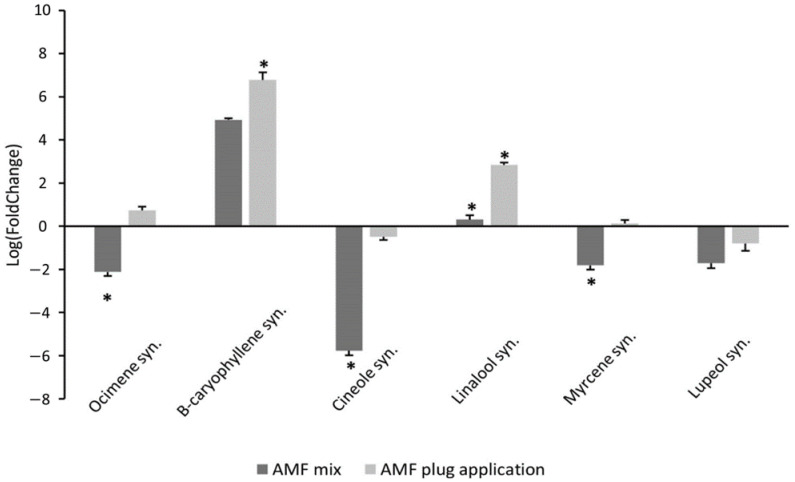
Change in gene expression of six terpene synthases in Rosemary ‘Perigord’ treated with different additions of AMF to the substrate. Fold change was calculated using ‘Perigord’ without AMF addition in peat substrate as control condition. Error bars are SEM of samples for each gene. * indicates a significant change versus untreated (*p* < 0.05 based on the Tukey HSD-test).

Terpene synthases play a key role in volatile and essential oil synthesis and their expression contributes to the aroma and taste of the plant. A range of terpene synthase genes were selected for analysis based on sequences previously identified in the rosemary genome sequence [6], which showed detectable transcripts when tested by qPCR. These represent a mix of mono-sesqi-and tri-terpene synthases responsible for terminal steps in synthesis of terpenes associated with rosemary volatiles and essential oil. The AMF root plug application showed statistically significant upregulation of expression of the genes, *β-caryophyllene synthase* and *Linalool synthase*, compared to the control condition (Figure 4). *β-caryophyllene synthase* showed a 32-fold increase in expression in rosemary ‘Perigord’ compared to the control (F = 151.3, *p* < 0.001). *Linalool synthase* also showed increased gene expression levels, by 4-fold, in the plug application method (F_2,10_ = 151.3, *p* < 0.01). The other four terpene synthases exhibited no significant change in expression with the AMF plug application method (*Ocimene synthase*, *Myrcene synthase*, *Lupeol synthase* and *Cineole synthase*).

Meanwhile, for the AMF treatment mixed into the substrate, there was an increased expression of *β-caryophyllene synthase*, by 9-fold, compared to the control plants (F_5,10_ = 151.3, *p* < 0.001, Figure 4). However, this change in levels of expression was significantly lower than in the direct application method with levels for the AMF mixture of only 0.28-fold of those observed for the plug application method (F_5,10_ = 151.3, *p* < 0.001). Other terpene synthases were downregulated with the addition of the AMF substrate mixture. *Cineole synthase* showed a large decrease with levels of 0.02-fold (F_5,10_ = 151.3, *p* < 0.001) compared to the control. *Ocimene synthase* and *Myrcene synthase* were both downregulated with levels of 0.29 and 0.17-fold, respectively, compared to the control (F_5,10_ = 151.3, *p* < 0.001).

### 3.2. AMF Substrate Mixture Alters Gene Expression of Terpene Synthases in Six Rosemary Cultivars

**Figure 5:**
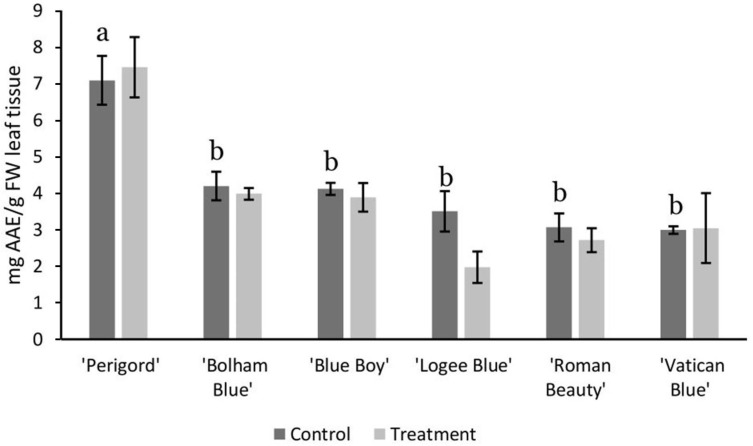
Antioxidant content (ascorbic acid equivalent, AAE) of the leaves of six different rosemary cultivars grown in peat substrate or treated with the addition of AMF. Error bars are SEM. Means in control conditions not sharing any letter are significantly different (*p* < 0.05 based on the Tukey HSD-test.

The mixture application method was then selected for further analysis of the impact of AMF supplementation on terpene synthase gene expression in additional cultivars. While both application methods showed good colonisation, the mixture application method was both less variable and provided a much more scalable approach, easily integrated with current practices within the horticultural industry and so this was chosen as the focus of our subsequent analysis. We assessed the effect of AMF addition to five additional rosemary cultivars, while also characterising variation between the cultivars themselves. There were considerable differences in height and width between the various cultivars, ‘Blueboy’ and ‘Vatican Blue’ had the tallest plants and were wider than ‘Perigord’. The shortest cultivar with a broader width was ‘Bolham Blue’ (Supplementary Figure S2). The addition of AMF did not affect the height or width of these cultivars, however, apart from ‘Logee Blue’ which showed an increase in plant height by an average 34.3% (F_1,4_ = 9.61, *p* < 0.05). Specialised metabolite production was assessed by measuring antioxidant content as an indicator of increased activity of specialised metabolism and potentially the improved production of volatile compounds. The commercial cultivar ‘Perigord’ had the highest antioxidant content of all the cultivars when grown in control conditions of peat substrate with antioxidant levels on average two-fold those observed in other cultivars (F_1,5_ = 35.11, *p* < 0.05, Figure 5). All other cultivars showed a consistent level of antioxidants with no substantial differences to each other. Overall, there was no significant difference between the antioxidant content of rosemary cultivars grown in peat substrate and those grown with addition of AMF. ‘Logee Blue’ treated with AMF showed 42.85% of the antioxidant content of control plants; however, this was not significant (F_1,5_ = 35.11, *p* > 0.05) based on the Tukey HSD-test.

**Figure 6:**
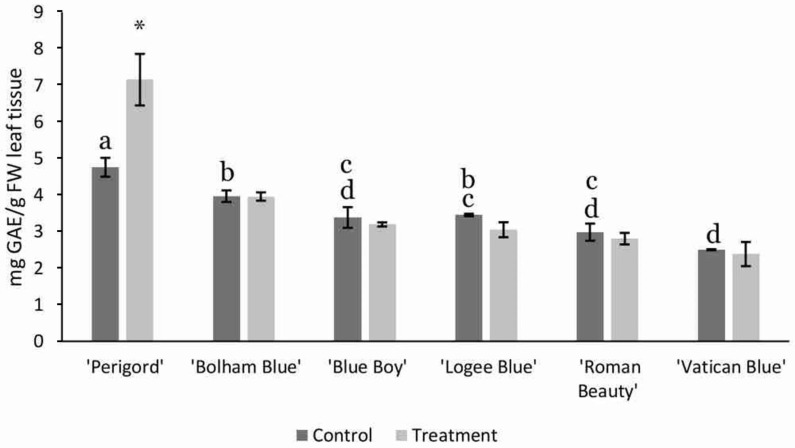
Total phenolic content (gallic acid equivalent, AAE) of six different rosemary cultivars grown in peat substrate containing AMF mixed in, compared with a control of peat substrate. Error bars are SEM. Means in control conditions not sharing any letter are significantly different; * represents significant difference between treated and control; (*p* < 0.05 based on the Tukey HSD-test).

The total phenolic content was also measured in the six cultivars. Under control conditions, there were several small but significant differences between cultivars. ‘Perigord’ had the highest phenolic content, while the cultivar ‘Bolham Blue’ also had a significantly higher phenolic content than ‘Blue Boy’, ‘Roman Beauty’ or ‘Vatican Blue’ (F_1,5_ = 2.21, *p* < 0.05, Figure 6). In addition, ‘Perigord’ showed a statistically significant (F1,5 = 2.21, *p* < 0.001) 1.5-fold increase in phenolic content when treated with AMF mix. However, the other rosemary cultivars showed no differences.

**Figure 7:**
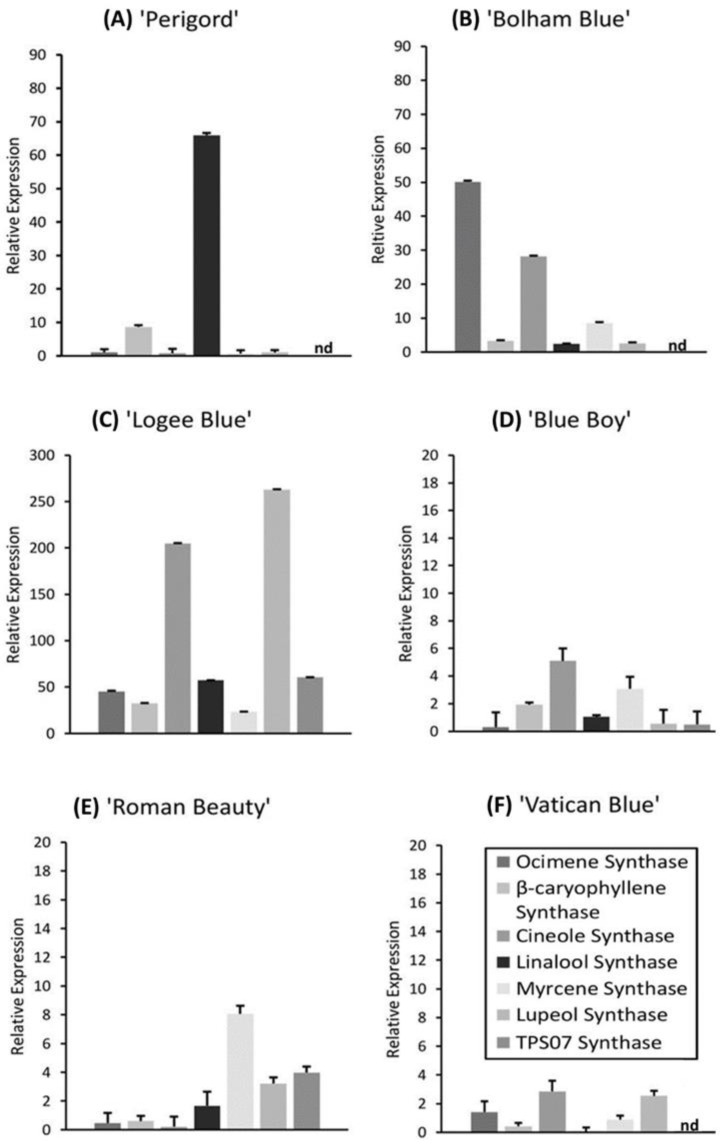
Cultivar comparison of relative expression levels of seven terpene synthases in different rosemary cultivars grown in un-supplemented peat substrate. The housekeeping gene GAPDH was used to calculate relative expression in each cultivar. No detection of expression represented by ‘nd’. Error bars are SEM.

The gene expression of seven terpene synthases was evaluated in the six cultivars of rosemary with and without the addition of AMF. The plants grown in peat substrate showed different relative expression levels between cultivars (Figure 7). ‘Logee Blue’ showed the highest overall levels of terpene synthase gene expression, while ‘Blue Boy’ and ‘Vatican Blue’ showed relatively low levels of all synthases. However, each cultivar had a unique gene expression profile in the control conditions. Notably, ‘Perigord’ showed relatively high levels of *Linalool synthase*; ‘Bolham Blue’ showed relatively high levels of *Ocimene* and *Cineole synthases*; ‘Logee Blue’ showed relatively high levels of *Cineole* and *Lupeol synthases*; while Roman Beauty showed relatively high levels of *Myrcene synthase*.

**Figure 8:**
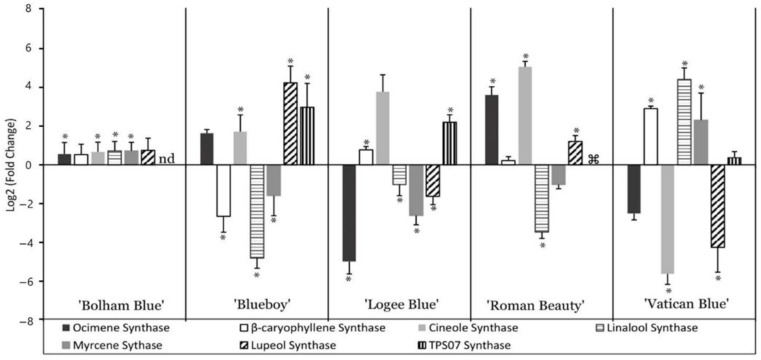
Change in gene expression of seven terpene synthases in five cultivars treated with AMF mixed into the substrate. Fold change was calculated by comparison of gene expression in each cultivar with controls in untreated peat substrate. * indicates a significant difference in gene expression between control and AMF conditions (*p* < 0.01 based on a Tukey HSD after ANOVA). nd: not detected in cultivar. ⌘: not detected following AMF treatment. Error bars are SEM.

The addition of the AMF mixture had varying effects on the expression of terpene synthases (Figure 8). ‘Bolham Blue’ showed small but significant increases in the expression of *Ocimene*, *Cineole*, *Linalool* and *Myrcene synthases* (F_5,24_ = 171.19, *p* < 0.001). ‘Blueboy’ showed a significant upregulation of *Cineole* and *Lupeol synthases* as well as an upregulation of *Terpene synthase 7* but a downregulation in *β-caryophyllene Linalool,* and *Myrcene synthases* when treated with AMF, while ‘Logee Blue’ showed significant increases in *β-caryophyllene synthase* and *Terpene synthase* 7 but a downregulation in *Myrcene, Linalool* and *Lupeol synthases* (F_5,24_ = 171.19, *p* < 0.001). When ‘Roman Beauty’ was treated with AMF, the cultivar showed an upregulation of *Ocimene, Cineole* and *Lupeol synthases* but downregulation of *Linalool synthase* (F_5,24_ = 171.19, *p* < 0.001). ‘Vatican Blue’ showed significant upregulation of *β-caryophyllene*, *Linalool* and *Myrcene synthases* and downregulation of *Ocimene*, *Cineole Lupeol synthases* (F_5,24_ = 171.19, *p* < 0.001). In all, this suggests that selection of variety and addition of AMF offer opportunities for significant modulation of aroma and flavour in rosemary.

## 4. Discussion

### 4.1. Root Colonisation Patterns Differed between the Two AMF Application Methods

The two AMF application methods trialled here both resulted in successful colonisation of the roots of rosemary plant cultivar, ‘Perigord’. In horticultural production, reducing the number of steps and resources to produce a high-quality plant product is highly desirable and the mixing of AMF into the substrate prior to planting plugs is more favourable in this respect than direct addition to the plug before planting. Fewer steps are involved in the AMF mixture method and so it would be easier to implement into current horticultural practices, making this method more favourable for commercial growers. A large batch of AMF substrate mixture can be easily prepared prior to transplanting. In contrast, with the direct application method a small, measured quantity of AMF is added to the location of every transplant before the plant is added to the pot. The latter would require an additional step to be integrated into the production line, reducing the efficiency of current horticultural practices.

None the less, there are some potential advantages to the plug application method. Rosemary plugs are young cuttings. When transplanted, their roots will take some time to grow and establish in the new substrate. With the direct application method to the root plug, the AMF is in higher concentration close to growing roots and, possibly, the AMF associate with the roots earlier as a result. This proposal is supported by the work of Scagel et al. [21] with Hick’s yew (*Taxus media*), a conifer which is also propagated by semi-hard wood cuttings. Arbuscular mycorrhizal fungi have been shown to improve the establishment of cuttings in Hick’s yew. The speed of root establishment is increased with the addition of AMF, indicating that mycorrhizal associations with young roots can significantly boost root growth. As a consequence, this will increase nutrients available to the plant in the early stages of growth [21]. Crucially, Scagel et al. [21] demonstrated that roots established more quickly, and the fungal-root cell interaction happened earlier when AMF was added directly to roots of young cuttings.

After root staining, differences in colonisation of the roots were observed between the AMF application methods. The percentage root length colonisation was higher in the direct application method than using the AMF mix method. However, the AMF plug application method had a more variable colonisation rate (as seen in Figure 1). The more uniform colonisation rates in plants treated with the AMF mixture will likely mean more consistent effects on the plants. Such consistency is desirable in pot grown herbs and would represent a further advantage of using this inoculation method over the plug application method in horticultural production.

### 4.2. AMF Application Enhanced Specialised Metabolism but Did Not Change Biomass or Morphology

Both application methods showed an increase in total phenolics in the commercial cultivar, ‘Perigord’. This could indicate that AMF stimulates the specialised metabolism including the MEP and MVA pathways for volatile production. The addition of arbuscular mycorrhizal fungi has been shown to boost the production of essential oils in rosemary cultivars [7,12]. Increasing the content of phenolic chemicals likely includes those that are associated with aroma and taste. As consumers choose fresh herbs based on a strong aroma, these changes to plant specialised metabolism would be beneficial. However, for cultivars other than ‘Perigord’, the addition of AMF made no significant difference to the quantity of phenolic compounds in the leaf extracts in our assay.

It is also important to note there was no morphological change after the AMF treatment for most of the rosemary cultivars. This could be due to conditions in horticultural glasshouses which are already fully optimised for growth. Other studies which have shown that AMF addition can provide a considerable boost to plant growth have generally studied growth in stressed conditions where growth is not already optimised [8,9,10,22]. This lack of a morphological response to AMF addition is, however, beneficial for commercial cultivars, which already have a desirable morphology for horticultural production and so no change to its morphology with treatment is desirable. This suggests that AMF addition is desirable for rosemary producers who wish to boost the specialised metabolism of the plants whilst retaining consistent morphological characteristics.

### 4.3. AMF Enhances Gene Expression of Terpene Synthases in Rosemary Cultivars

The gene expression analysis showed that there were considerable differences in terpene expression between control and AMF treated rosemary plants. It also showed that the cultivars had different patterns of terpene synthase expression without the AMF addition. ‘Logee Blue’ is reported to be a particularly aromatic cultivar compared to the others [23], which may be due to the high expression levels of seven terpene synthases observed in these plants. Responses to the AMF mixture also vary among cultivars. A general upregulation of terpene synthases was seen in ‘Bolham Blue’ which had increases in all six of the terpene synthases detected. In other cultivars, responses varied, with large increases often seen in just one or two terpene synthases. Patterns of up and downregulation varied greatly between cultivars; though, there was some commonality between certain cultivars. For example, both ‘Blue Boy’ and ‘Roman Beauty’ both showed increases in *Cineole* and *Lupeol synthases* and downregulation of *Linalool synthase*. Likewise, both ‘Logee Blue’ and ‘Vatican Blue’ showed upregulation of *β-caryophyllene synthase* and downregulation of *Lupeol synthase*. For the grower, these results show that cultivar selection is relevant not only when selecting for increased aromatics but also when considering application of AMF. As rosemary cultivars have demonstrated here a wide variation in responses in terpene synthase gene expression, this implies that a similar variation might be expected in terms of aroma.

Prediction of details of aroma changes based purely on gene expression analysis is quite unreliable as the final aroma is a result of the blend of volatiles produced [24]. However, some predictions based on individual pathway end products may be attempted. For ‘Perigord’, the AMF addition enhanced the gene expression of some key terpene synthases. *β-caryophyllene synthase* was upregulated, along with *Linalool synthase*. The upregulation of *β-caryophyllene synthase* could lead to an increase in synthesis of two sesquiterpene products, β-caryophyllene and α-humulene [25]. These are associated with a peppery clove smell and a woody, slightly bitter odour, respectively. The upregulation of *Linalool synthase* would also be likely to alter the aroma profile of ‘Perigord’ since Linalool is a main constituent of rosemary essential oil. It contributes to the aroma profile with an aroma of pine and floral notes. This may be a beneficial enhancement of the aroma profile of ‘Perigord’ as consumers may find this rosemary more fragrant. *Ocimene synthase* is responsible for the synthesis of β-ocimene, terpinolene, β-myrcene, and β-pinene, all constituents of rosemary volatiles and essential oils. This synthase was upregulated in ‘Bolham Blue’, ‘Blue Boy’ and ‘Roman Beauty’, indicating that AMF may be enhancing the quality of these aromatics in these cultivars. However, there were some notable decreases that should be considered. For example, ‘Blue Boy’ and ‘Logee Blue’ showed decreases in *Myrcene synthase*, with addition of AMF. This is responsible for the synthesis of myrcene, which has a peppery aroma [24].

Most frequently, addition of AMF led to an increase in expression of *Cineole synthase*, a phenomenon observed in cultivars ‘Bolham Blue’, ‘Blueboy’, ‘Logee Blue’ and ‘Roman Beauty’. That would likely result in an increase in monoterpenes with strong woody, spicey, and floral scents.

Control of gene expression in metabolic pathways often involves complex regulatory systems. It is possible that specific changes in gene expression may be triggered by chemical communication between the AMF and the plant [26]. Equally, they may be triggered by changes in levels of an enzyme’s substrate leading to increased enzyme production [27]. In the case of AMF, such changes in substrate could result from improved availability of nutrients resulting in a change in the flux of metabolites through other connected biosynthetic pathways. For example, since sesquiterpenes are synthesised using the MVA pathway [28], it is possible that AMF addition is altering specialised metabolite production at an earlier stage of the MVA pathway. A possible mechanism for this is the AMF providing additional substrates and nutrients, such as phosphorus [29], for the MVA pathway, thereby increasing the availability of the precursors, DMAPP and IPP, for terpene synthases. In support of this, a previous study has shown that phosphate fertilizers improve essential oil yield and linalool quantities in lavender [30]. Therefore, AMF could be enhancing terpene synthesis through the additional nutrient uptake provided to the plant. It has also been demonstrated that microbial supplementation, specifically, using plant growth-promoting bacteria, can reduce the impact of environmental stress on plants, altering antioxidant levels [31]. It is possible that AMF addition could also be acting in this case via alteration of plant stress responses which may influence terpene synthase expression. Finally, it is important to note that the exact impact of AMF addition may vary depending on the initial microbial content of the substrate. The magnitude and even direction of the effect may be impacted by interaction of the initial substrate microbial community with the AMF. However, our key conclusion is that AMF addition offers a treatment which can be used with potted rosemary, grown in short term cultivation in a horticultural setting, to potentially alter volatile production and, therefore, aroma.

## 5. Conclusions

We have used rosemary as a model to investigate the potential for AMF to modify pathways involved in synthesis of volatile aromatic compounds in potted herbs. We investigated the impact of AMF addition on expression of several genes encoding key enzymes within volatile biosynthetic pathways in rosemary. Our findings show that a more consistent colonisation of rosemary roots was achieved when AMF was mixed into the substrate prior to planting of root plugs as opposed to being locally applied as the plug was planted. AMF application did not significantly affect the biomass or morphology of plants in a commercial setting but did alter gene expression values of terpene synthases in all cultivars tested. Significant varietal differences were observed between cultivars both in terms of basal gene expression values and in terms of response to AMF addition. The absence of an effect on biomass and morphology is of benefit to the commercial potted rosemary industry, where parameters of uniformity and height are already optimised for large scale cultivation and shipping to supermarkets. However, by revealing an extensive reprogramming of terpene synthase gene expression in response to AMF, in a range of rosemary cultivars, we demonstrate the potential for AMF addition in a commercial setting to significantly alter the aromatic profile of the plants. Overall, our findings demonstrate that AMF addition to commercial pot-grown herbs has the potential to enhance the aroma and taste, while maintaining consistency of plant shape and visual aspects.

## Figures and Tables

**Table 1:** qPCR primers for selected terpene synthases, showing sequence of Forward and Reverse primers, their temperature ranges, G-C% content and product length in bp.

Gene Name	Arabidopsis Ortholog	Forward Primer	Reverse Primer
*Glyceraldehyde 3-phosphate dehydrogenase*	GAPDH	AAGCATCGGAGACCAAGCTC	CGCGAGAACTGTAACCCCAT
*Ocimene synthase*	TPS03	GGTACCACACGGGGCATAAA	CAAGATCATCTGCAAGCCGC
*β-caryophyllene synthase*	TPS12	AGACTGGCCGTAGCAAACTC	CCGATTGTTCAGGCAACACG
*Cineole synthase*	TPS27	CAGGCATCCTTGCCACATGA	GCCAAACGTTGAGAAAGCCC
*Linalool synthase*	TPS14	GCCAAATTCAGAGAGGCCCTT	TTGTCCGAGAAGGAAGCACG
*Myrcene synthase*	TPS24	TGACGCGAACCCTATTCTGG	CAAACCCCAACTTTTCCGGC
*Lupeol synthase*	LUP2	CTGGCTCTTCCCTTCCGTTT	TAAAACGACGTCGGTGAGGG
*Terpene synthase 07*	TPS07	CGATGTTCGTGTTCTTGCCC	CCTTCAAATCTCCTCCCCCG

## Data Availability

Not applicable.

## Supplementary Materials

The following supporting information can be downloaded at: https://www.mdpi.com/article/10.3390/life13020315/s1, Figure S1: Rosemary cultivar, Perigord, after 9 weeks growth in a glasshouse following transplant of an initial the root plug (A) into untreated substrate, (B) into substrate in which AMF mixture was mixed, and (C) into untreated substrate following AMF addition to the surface of the initial root plug; Figure S2: Height and width measurements of potted rosemary varieties treated with AMF.
