# Supplementary material for: Addition of Arbuscular Mycorrhizal Fungi Enhances Terpene Synthase Expression in Salvia rosmarinus Cultivars
Source: Life (Basel). 2023 Jan 23;13(2):315. doi: 10.3390/life13020315 (PMC9959559; doi:10.3390/life13020315)
Supplement: Supplementary file 1 [file life-13-00315-s001.zip › life-2150327-supplementary.pdf]

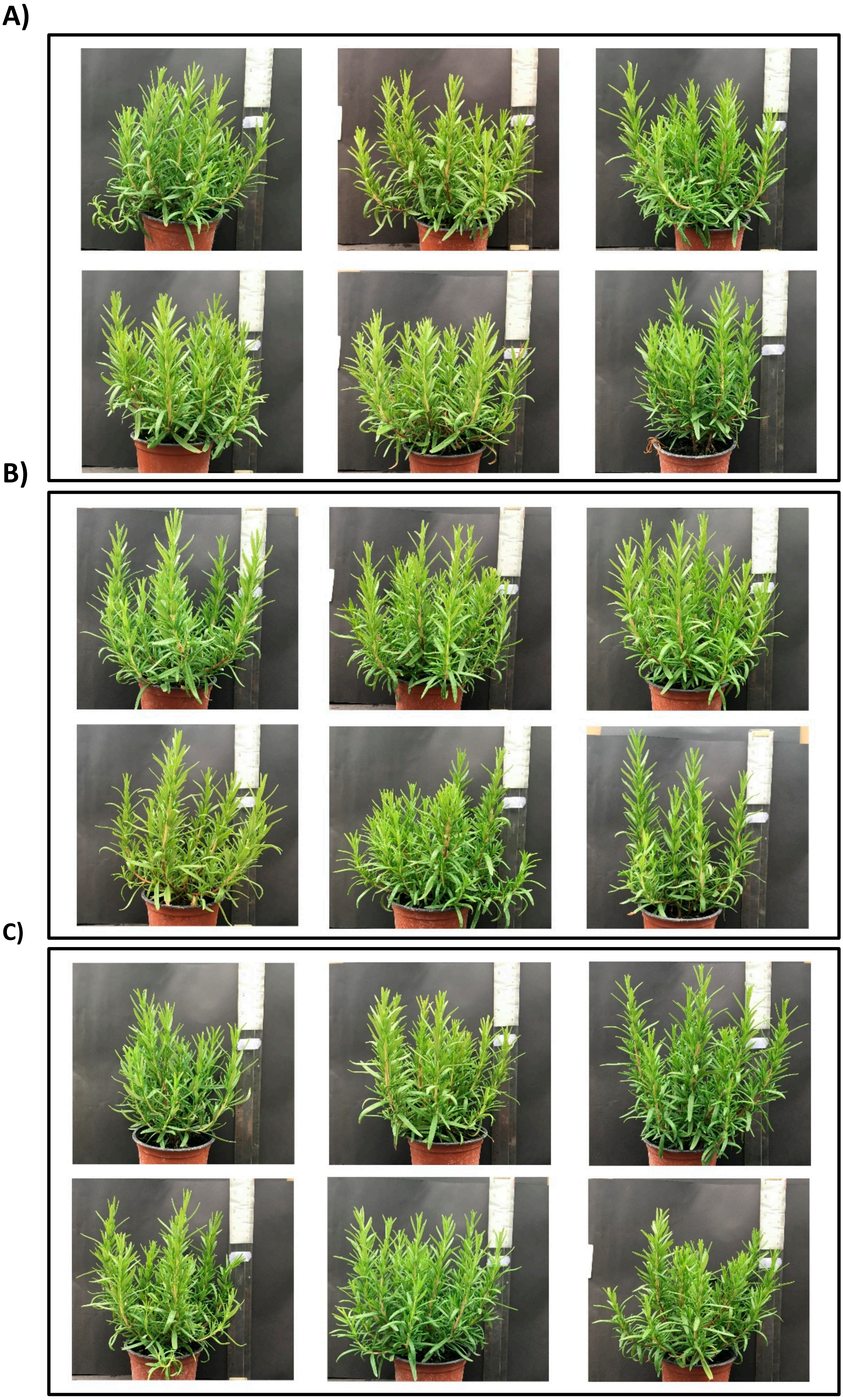

Supplementary Figure S1. Rosemary cultivar, Perigord, after 9 weeks growth in a glasshouse following transplant of an initial the root plug A) into untreated substrate, B) into substrate in which AMF mixture was mixed, and C) into untreated substrate following AMF addition to the surface of the initial root plug.

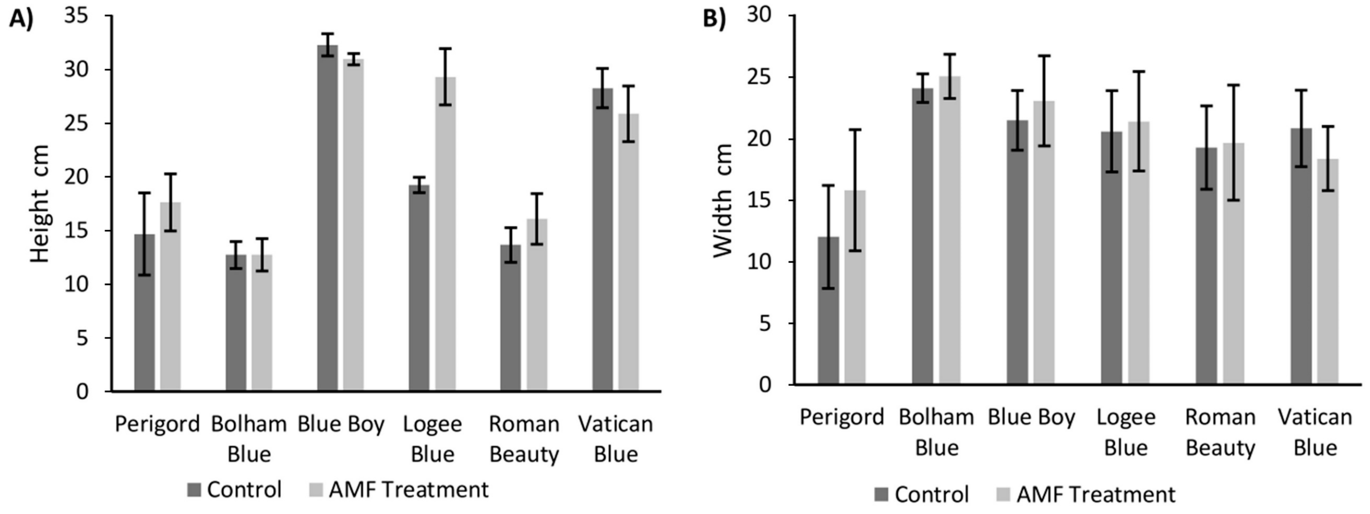

Supplementary Figure S2. Height and width measurements of six different rosemary cultivars grown in peat substrate or treated with the addition of AMF. Error bars are SEM. A) height, B) width. Error bars are SEM.
